# Clinical experience with intravenous administration of ascorbic acid: achievable levels in blood for different states of inflammation and disease in cancer patients

**DOI:** 10.1186/1479-5876-11-191

**Published:** 2013-08-15

**Authors:** Nina Mikirova, Joseph Casciari, Neil Riordan, Ronald Hunninghake

**Affiliations:** 1 Riordan Clinic, 3100 N. Hillside, Wichita, KS 67219, USA

## Abstract

**Background:**

Ascorbic acid (vitamin C, ascorbate) is a key water soluble antioxidant that, when administered in doses well above its recommended dietary allowance, may have preventative and therapeutic value against a number of pathologies. The intravenous administration of high dose ascorbate (IVC) has increased in popularity among complementary and alternative medicine practitioners: thousands of patients received IVC, at an average dose of 0.5 g/kg, without significant side effects. While IVC may have a variety of possible applications, it has generated the most interest for its potential use in treating cancer.

**Methods:**

Medical records of patients with cancer treated with IVC at the Riordan Clinic were retrospectively reviewed. Cancer patients, for whom plasma ascorbate concentration data before and after treatment were available, along with C-reactive protein (CRP) measurements, were chosen for analysis.

**Results:**

The results of the analysis can be summarized as follows. IVC produces peak plasma ascorbate concentrations on the order of ten millimolars with lower peak plasma concentrations obtained in cancer patients as compared to healthy subjects. Cancer patients who are deficient in vitamin C prior to therapy tend to achieve lower plasma levels post infusion. High inflammation or tumor burdens, as measured by CRP or tumor antigen levels, tend to lower peak plasma ascorbate levels after IVC. When compared to patients with localized tumors, patients with metastatic tumors tend to achieve lower post infusion plasma ascorbate concentrations.

**Conclusions:**

The data indicate that, while potentially therapeutic plasma ascorbate concentrations can be achieved with IVC, levels attained will vary based on tumor burden and degree of inflammation (among other factors). Evidence suggests that IVC may be able to modulate inflammation, which in turn might improve outcomes for cancer patients. IVC may serve as a safe, adjunctive therapy in clinical cancer care.

## Background

Vitamin C is an antioxidant that increases extracellular collagen production and is important for immune cell functioning [1,2]. The intravenous administration of vitamin C involves the slow infusion of vitamin C at doses on the order of 0.1 to 1.0 grams per kilogram body mass and has become increasingly popular among complementary and alternative medicine practitioners [3]. When vitamin C is given by intravenous infusion, peak concentrations over 10 mM, two orders of magnitude above what is observed with oral supplementation, can be attained [4,5] without significant adverse effects to the recipient. While IVC may have a variety of possible applications, such as combating infections [6-8], treating rheumatoid arthritis [9], it has generated the most interest for its potential use in treating cancer.

Vitamin C can potentially help cancer patients in a variety of ways: its role in collagen production may protect normal tissue from tumor invasiveness and metastasis [10,11], while vitamin C replenishment in cancer patients, who are often depleted of this vitamin [12,13], may improve immune system function and enhance patient health and well-being [14].

The use of high doses intravenously has drawn particular interest for the following reasons:

• A profound reduction of plasma ascorbate levels is observed in cancer patients [15-21]. This ascorbate deficiency (clinical scurvy) was correlated with elevated levels of the inflammation marker C-reactive protein often manifesting the shorter survival times.

• At concentrations on the order of 1 mM, ascorbate can cause a build-up of hydrogen peroxide, which is preferentially toxic toward tumor cells [4,22,23]. Experimental studies confirm that ascorbate concentrations sufficient for this cytotoxic effect can be attained in vivo, and that treatments can reduce tumor growth in animal models [24-28].

• Ascorbate, at concentrations of 1 to 10 mM, can have an inhibitory effect on tumor angiogenesis [29-34], a process of new blood vessel formation that is considered critical to tumor growth and metastasis.

Phase I clinical trials indicate that IVC can be administered safely with relatively few adverse effects [13,35]. Clinical studies have demonstrated that IVC significantly improved global quality of life scores in cancer patients. Patients given IVC in addition to standard oncologic treatments benefited from less fatigue, reduction in nausea, improved appetite, reductions in depression and fewer sleep disorders [36,37], and their overall intensity scores of adverse symptoms during therapy and aftercare were half those of the control (no IVC) group. Other studies report anti-cancer efficacy, improved patient well-being, and decreases in markers of inflammation and tumor growth [38-43].

The relationship between IVC dose and plasma ascorbate concentration is important in understanding the ascorbic acid’s effect on cancer. In this regard, we analyzed this relationship in a large database of cancer patients given IVC therapy; moreover, we examined the dependence of plasma ascorbate concentrations in patients with localized and metastatic tumors on C-reactive protein levels and tumor marker levels.

## Methods

The biochemical assays and analysis were performed at the Riordan Clinic Laboratory. CRP concentrations in blood (serum or heparin-plasma) were determined using a particle-enhanced immune-turbidimetric assay (CRP Ultra WR Reagent kit, Genzyme) according to manufacturer’s instructions on an automated analyzer [CobasMIRA, Roche Diagnostics]. The upper boundary for the normal range was set to 1.9 mg/L. Vitamin C was measured by high-pressure liquid chromatography (HPLC) with electrochemical detection.

Measurements of tumor antigen levels were carried out by Lab Corp. For CA15-3, Ca29.29, and CA125 electrochemiluminescence immunoassays (ECLIA) were used.

The study was conducted under Institutional Review Board Approval of Riordan Clinic, Wichita, KS, USA. Demographics were limited to ensure confidentiality.

From the database of cancer patients treated with IVC at the Riordan Clinic, we selected subjects for whom plasma ascorbic acid levels before and after treatment were available along with laboratory tests of inflammation marker CRP and cancer markers. A breakdown of cancer types for these subjects along with sex, age, weight ranges, and average plasma ascorbate levels before and after the first IVC infusion is provided in Table 1.

**Table 1:** Characteristics of cancer patients from the clinic database selected for data analysis: number of subjects, sex, age range, weight range, and average ascorbate concentrations before and immediately after the first 15 gram IVC infusion

**Cancer type**	**N**	**M/F**	**Age (years)**	**Weight (lbs)**	**Ascorbic acid, post (mM)**	**Ascorbic acid, pre (mM)**	**Ascorbic acid, minimum pre (mM)**
Bladder	10	8/2	32–80	160–192	5.00	0.078	0.045
Brain	12	3/9	26–66	126–137	6.73	0.051	0.045
Breast	105	1/133	38–72	104–190	6.57	0.076	0.023
Breast, metastatic	28	0/28	36–64	93–250	5.45	0.060	0.017
Chronic Lymphocytic Leukemia	15	8/7	50–67	125–213	5.52	0.059	0.023
Colon	34	23/11	50–78	110–280	5.46	0.063	0.017
Colon, metastatic	26	16/10	50–69	106–200	6.23	0.062	0.028
Esophagus	5	5/0	48–77	ND	5.99	0.080	0.068
Esophagus, metastatic	5	5/0	50–63	114–280	6.11	0.055	0.011
Liver	8	5/3	14–58	99–157	5.78	0.057	0.034
Liver, metastatic	7	3/4	50–69	85–160	5.90	0.052	0.028
Lung	43	25/18	49–75	104–290	5.81	0.051	0.011
Lung, metastatic	20	10/10	25–78	107–178	5.07	0.060	0.017
lymphoma (non-Hodgkin’s)	6	1/5	40–65	120–159	5.13	0.077	0.063
Melanoma	11	3/8	26–72	130–207	5.26	0.068	0.063
Ovarian	36	0/36	31–76	108–217	6.59	0.058	0.017
Pancreas	16	11/5	58–80	149–200	5.65	0.051	0.028
Pancreas, metastatic	11	7/4	65–80	132–174	5.13	0.068	0.045
Prostate	64	64/0	59–89	147–250	5.61	0.063	0.017
Prostate, metastatic	7	7/0	59–88	150–225	4.64	0.059	0.023
Renal	17	10/7	51–76	72–229	6.08	0.055	0.040
Renal, metastatic	13	7/6	39–68	ND	5.89	0.063	0.040
Sarcoma	7	5/2	21–75	90–163	4.98	0.045	0.023
Skin	6	6/0	54–76	175–211	5.54	0.051	0.028
Stomach	5	4/1	33–77	ND	4.75	0.051	0.028
Throat	7	4/3	34–80	ND	5.40	0.063	0.034
Thymus	7	4/3	48–73	48–73	5.39	0.068	0.051
Uterus	7	0/7	52–70	99–173	6.62	0.068	0.051

The details of the Riordan IVC protocol have been described elsewhere [44]. Briefly, new cancer patients are given a 15 gram injection for their first dose, followed by a 25 gram injection the next day. Dosage is then adjusted by the physician based on the patients’ tolerance and plasma ascorbate levels attained post infusion.

## Results

**Figure 1:**
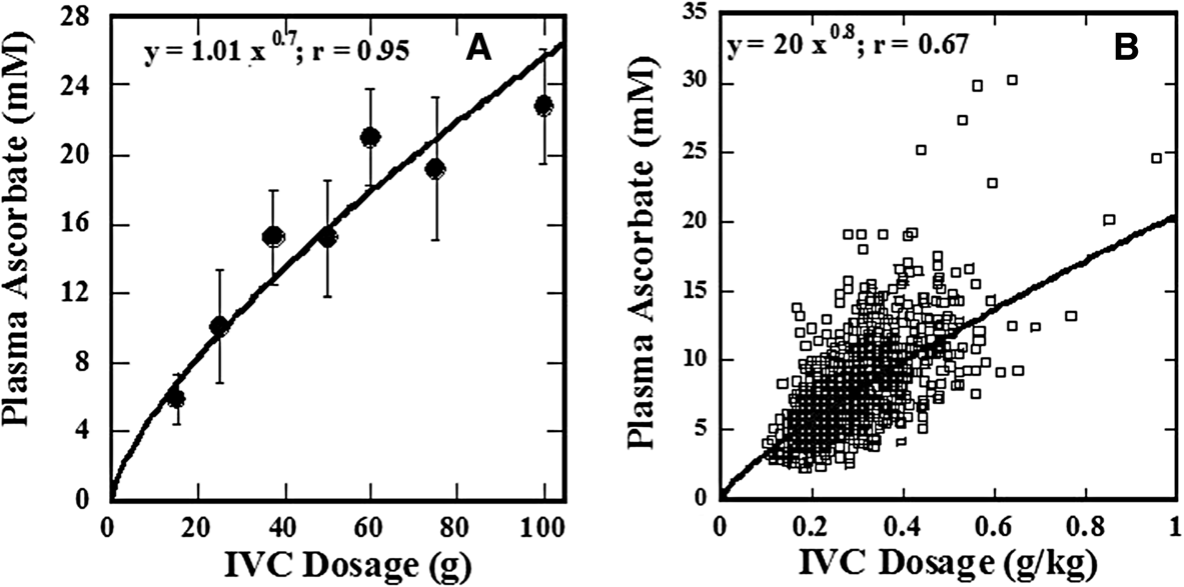
**The peak plasma ascorbic acid concentration as a function of IVC dosage. A)** The peak plasma ascorbate concentration averaged for all subjects**B)** Plasma concentration as a function of ascorbate dose per kg body mass.

From Table 1, it can be seen that intravenous ascorbate infusions of 15 grams increase plasma ascorbate levels by one or two orders of magnitude. The average pre and post 15 g IVC concentrations of ascorbate in blood were 0.06 ± 0.01 mM and 5.7 ± 0.6 mM. Figure 1 illustrates how the peak plasma ascorbate level is affected by the IVC dosage used. Consequently, higher doses provide higher plasma concentrations, but the effect is not linear. This is probably because IVC is administered as a slow “drip” over a dose-dependent time period (15 grams are administered over 0.5 hours, while 100 grams are administered over a 3.5 hours), giving the body more time to clear some of the ascorbate through the kidneys at higher doses. As Figure 1B in particular shows, there is variability in the plasma levels attained, even if the dosage is normalized to body mass. It is important to monitor plasma levels for individual patients, as the pharmacokinetics may vary considerably from person to person.

**Figure 2:**
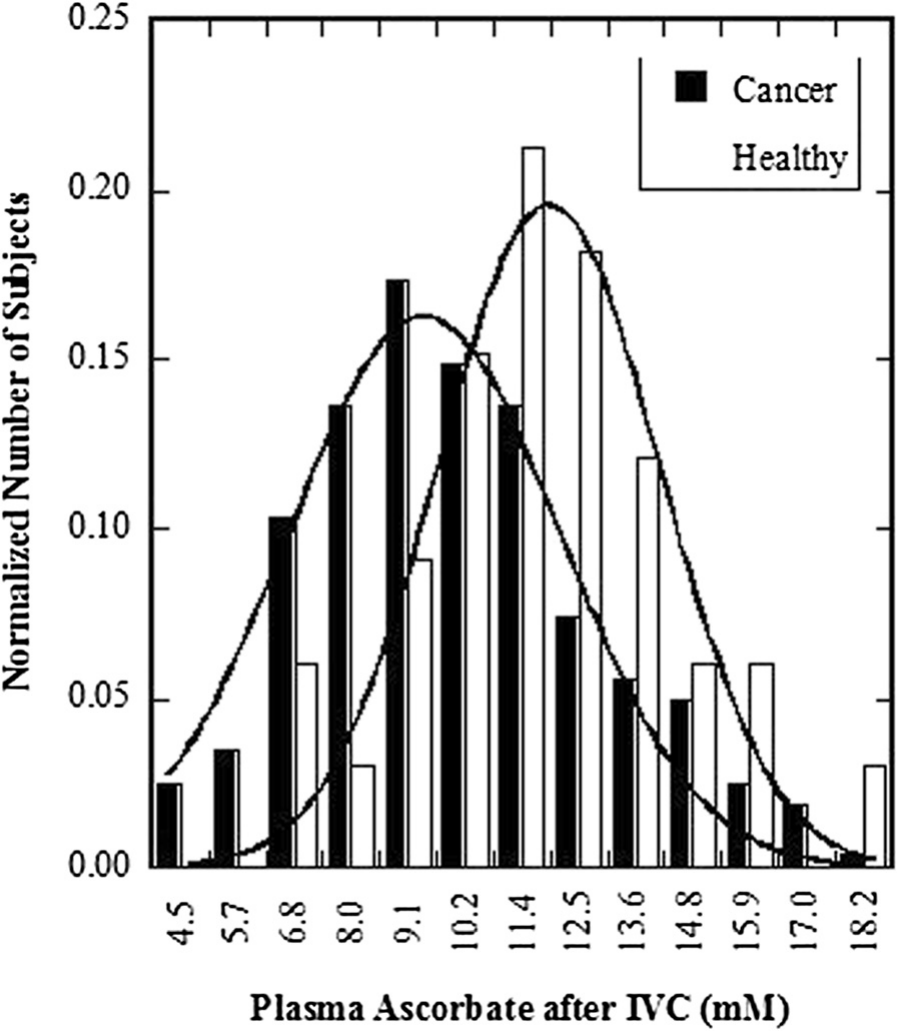
**Distribution of peak plasma ascorbate concentrations in healthy adults and cancer patients.** Curve fits are Gaussian.

Plasma levels after IVC infusion tend to be lower in cancer patients relative to healthy adults. This is illustrated in Figure 2, where the distributions of ascorbate concentrations for cancer patients and healthy adults given 25 grams IVC are shown.

This suggests that cancer patients may need higher doses to achieve a given plasma concentration. We found that there was a weak but statistically significant correlation between the pre-treatment plasma ascorbate concentration and the post-IVC plasma concentration attained (r = 0.28, N = 193) suggesting that patients with lower vitamin C levels may see more distribution of intravenously administered ascorbate into tissues and thus attain less in plasma. When treating patients with IVC, the first treatment likely serves to replenish depleted tissue stores, if those subjects were vitamin C deficient at the beginning of the treatment. Then, in subsequent treatments, with increasing doses, higher plasma concentrations can be attained. On-going treatments serve to progressively reduce oxidative stress in cancer patients.

**Figure 3:**
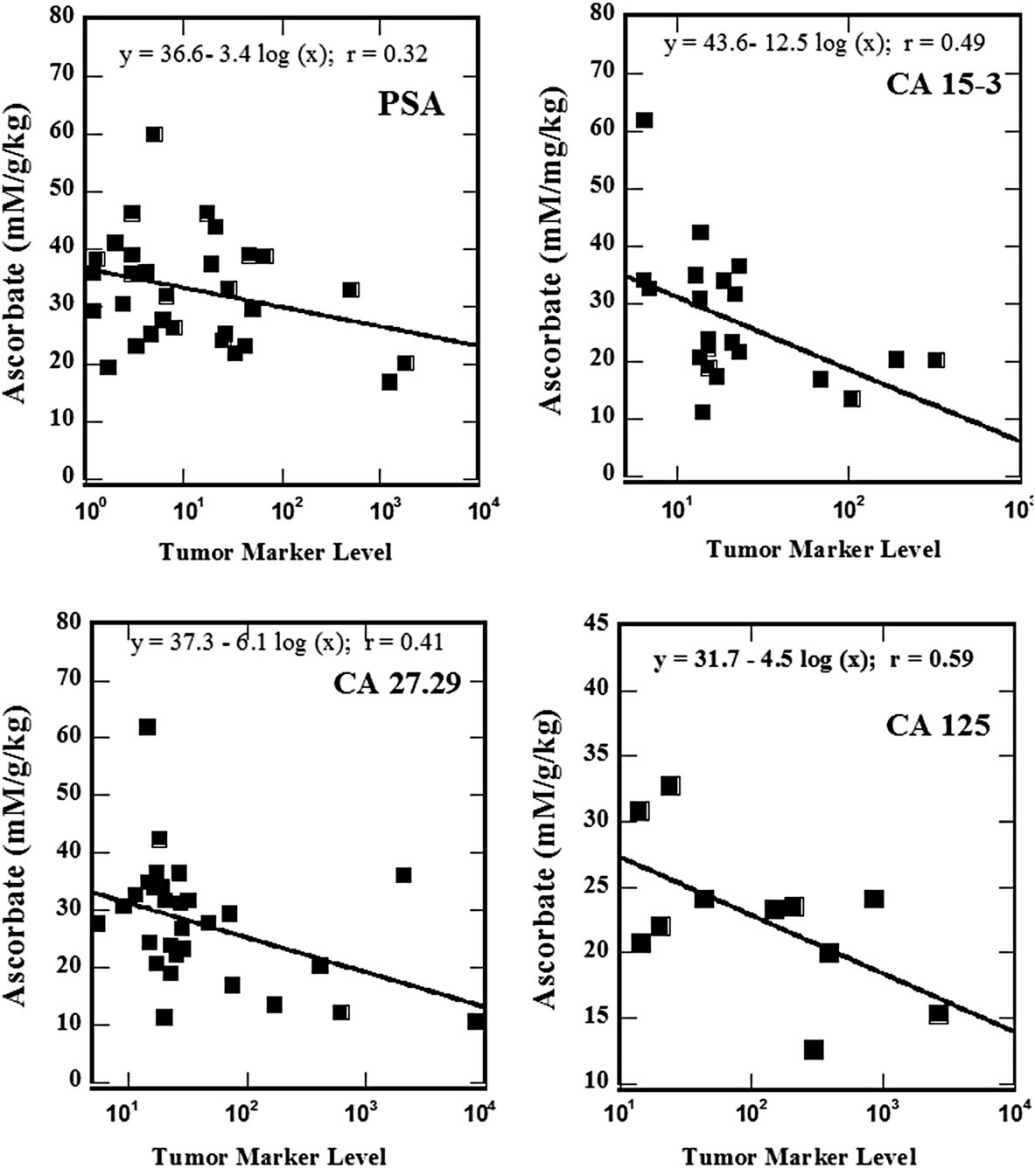
**Relative plasma ascorbic acid concentrations (mM divided by dose in grams per kg weight) as a function of tumor marker values for PSA, CA 15–3, CA 27.2, CA 125, CEA, and the inflammation marker CRP.** Data fits are y = a + b log(x); r values for CA 15–3, CA 27.29, CA 125, and CRP are significant for correlation at the 95% confidence level (p < 0.05) while those for PSA and CEA are significant at the 90% level (p < 0.10).

**Figure 4:**
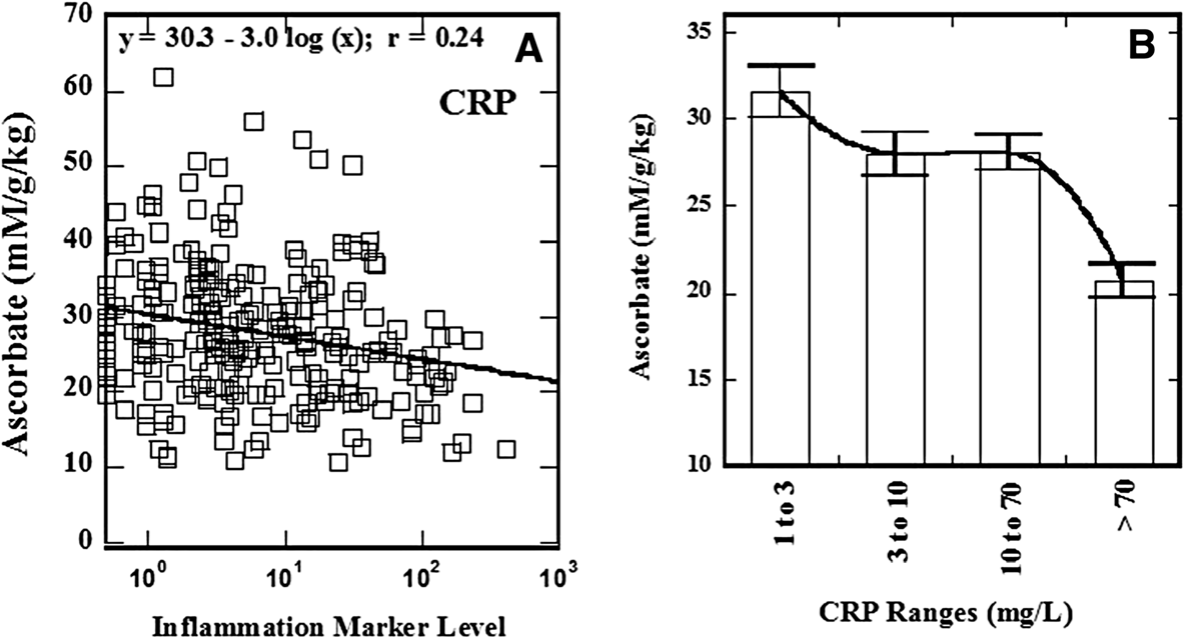
**Dependence of the achievable levels of ascorbic acid in blood on inflammation. (A)** Plasma ascorbate concentrations in mM divided by dose in grams per kg weight as a function of CRP and **(B)** mean values of the relative plasma ascorbic acid in four groups of patients sorted based on CRP levels. Error bars are given as standard errors.

Figures 3, 4 show how plasma ascorbate levels (after first IVC infusion) correlated with the expression of key tumor and inflammation markers. We examined the prostate antigen PSA, the breast cancer markers CA 27.29 and CA15-3, the ovarian cancer marker CA 125, the general cancer marker CEA, and the inflammation marker C – reactive protein.

The data presented in Figure 3 show the tendency of lower achievable plasma levels of vitamin C at higher levels of tumor markers. Patients with higher tumor markers are likely to have higher tumor burden, higher oxidative stress and, therefore, are more likely to have lower post IVC plasma levels.

This also seems to be the case for patients with elevated inflammation, measured by CRP. Figure 4A illustrates the relationship between inflammation and ascorbate pharmacology. Data are plotted as plasma ascorbate (plasma concentration in mM divided by dose in g/kg) versus the inflammation marker CRP. Values for CRP are significant for correlation at the 95% confidence level (p < 0.05).

In addition, patients were divided into four groups based on CRP levels, and the average plasma ascorbate concentration normalized to dose (mM per g/kg) for each group was calculated (Figure 4B). The patients who showed most severe inflammation (CRP > 70 mg/L) had significantly lower plasma ascorbate levels after infusion (p < 0.01).

**Figure 5:**
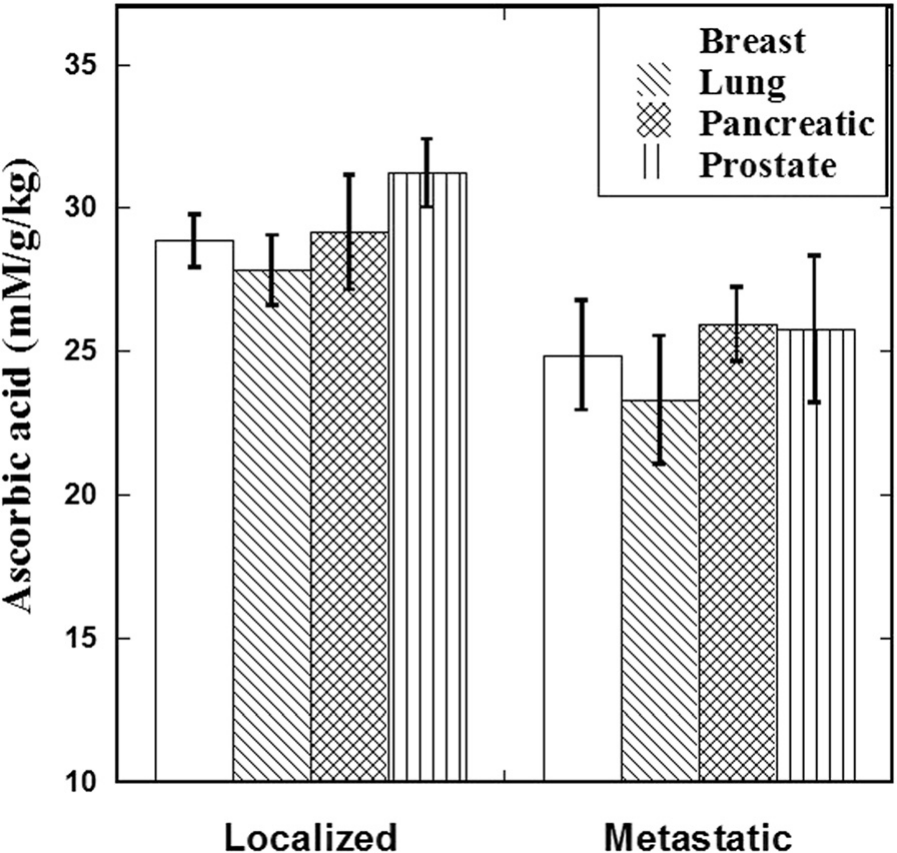
Mean values of the relative plasma ascorbate (concentration in mM divided by dose in g/kg) in patients with various types of localized or metastatic cancer after their first 15 gram IVC infusion.

Our data also showed that cancer patients with metastasis tend to have lower post-IVC vitamin C levels than those without metastasis (Figure 5).

These pharmacokinetic data can be summarized up as follows: IVC produces peak plasma ascorbate concentrations (30–50 min after beginning of infusion) on the order of ten millimolars. Results are highly variable from patient to patient, with the following tendencies observed:

• Lower peak plasma concentrations are obtained in cancer patients than in healthy subjects. Cancer patients who are deficient in vitamin C prior to therapy tend to achieve lower plasma levels post infusion.

• Patients with higher inflammation or tumor burdens, as measured by CRP levels or tumor antigen levels, tend to show lower peak plasma ascorbate levels after IVC.

• Patients with metastatic tumors tend to achieve lower post infusion plasma ascorbate levels than those with localized tumors.

**Figure 6:**
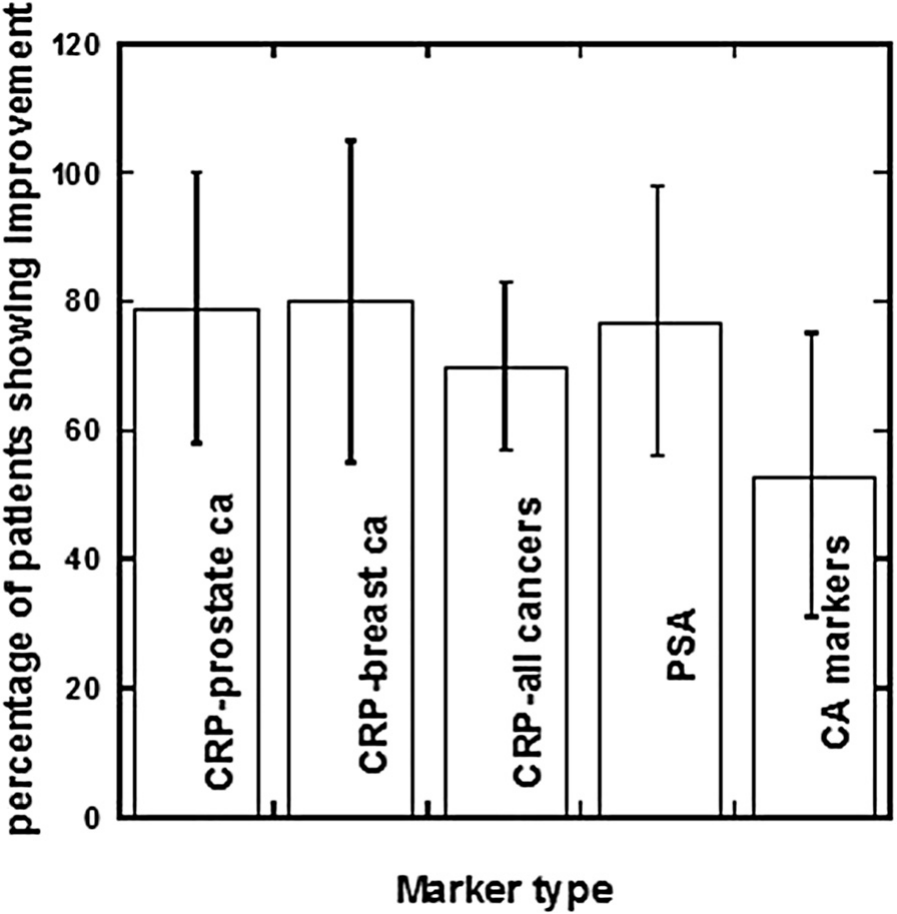
Changes in key parameters for cancer patients after IVC therapy.

We also used the Riordan Clinic database to determine if tumor and inflammation markers were affected by long term IVC therapy. The detailed characteristics of subjects under analysis with duration of treatment, numbers of IVCs, and inflammation markers before and after treatments were presented in our publication [42]. We were able to analyse data from forty-eight patients, with a mean follow-up time of seven years. The median age of the patients was 68 years, with a range of 47–85 years. Roughly half of the patients had prostate cancer while twenty percent had breast cancer and the rest of the patients had liver, pancreatic, bladder and skin cancers. Number of treatments ranged from three to 100, at a frequency of one or two treatments per week. Figure 6 shows the proportion of patients who had reductions in tumor or inflammation markers. Regarding inflammation, 73 ± 13% of subjects (95% confidence) showed a reduction in CRP levels during therapy. This was an even more dramatic 86 ± 13% (95% confidence) in subjects who started therapy with CRP levels above 10 mg/L. In these subjects, the median reduction in CRP level was 80%, with the IQR being 39% to 94%.

Most of the prostate cancer patients studied, 75 ± 19% (95% confidence), showed reductions in PSA levels during the course of their IVC therapy (Figure 6, Table 2). The data in Table 2 show the characteristics of patients with prostate cancer, values of PSA before and after treatment, numbers of treatments, and duration of treatments and percentage of improvement in PSA values.

**Table 2:** The characteristics of patients with prostate cancer from the clinic database with values of PSA before and after treatment, number of treatments, duration of treatments and percentage of improvement

**Type of cancer**	**Grade, score**	**PSA before**	**PSA after**	**Days of treatment**	**Number of treatments**	**Percentage of improvement**
Prostate	Gleason score - 6-9	1507	46	531	98	96.9
Prostate	Grade II-III; Stage B2	116	3.7	302	7	96.8
Prostate	ND	788	21.1	112	15	97.3
Prostate	Gleason score 2.5	54	12.3	343	32	77.2
Prostate	ND	35.8	1	89	10	97.2
Prostate	Gleason 7	48.8	5.8	4863	18	88.1
Prostate	Gleason score 6	44	2.7	161	20	93.9
Prostate	Gleason score 9	5.1	71.2	143	33	−1296.1
Prostate	ND	65	184	588	13	−183.1
Prostate	Gleason score 4	26	5.5	146	5	78.8
Prostate	Gleason score 4	63	0.5	112	7	99.2
Prostate	Gleason score 5	20	2.3	214	6	88.5
Prostate	ND	153	87	247	4	43.1
Prostate	ND	7.3	2.3	136	8	68.5
Prostate	Gleason score 6	11	8	887	42	27.3
Prostate, metastatic	ND	51.3	21	500	32	59.1
Prostate, metastatic	ND	21	37.1	139	52	−76.7
Prostate	Gleason score 8	8	0.1	481	28	98.8
Prostate	ND	14.6	36.2	1652	81	−147.9
Prostate	Gleason score 6	11	29	1000	11	−163.6
Prostate, metastatic	ND	28.6	86.9	89	24	−203.8
Prostate	Gleason score 6-8	48.8	1.2	121	15	97.5
Prostate	Gleason score 6	65.2	13.8	45	4	78.8
Prostate	Gleason score 6-8	106	86.5	310	11	18.4
Prostate, metastatic	Stage II	500	54.3	542	77	89.1
Prostate	Gleason score 6.5, stage II	10.6	7.5	643	27	29.2

In subjects with detailed data of inflammation and tumor markers, we analyzed the correlation between changes in CRP levels and changes in tumor markers after IVC therapy. The data are presented in Table 3. In those cases, there was a strong correlation (R ^2^ = 0.6) between the change in tumor marker and the change in CRP during IVC therapy.

**Table 3:** Relation between changes in CRP levels and changes in tumor markers (TM) after IVC therapy

**Type of cancer**	**% of CRP change**	**% of TM change**	**Type of cancer**	**% of CRP change**	**% of TM change**
Prostate	82.5	99.7	breast	−23.3	−46.7
Breast	80.0	21.3	prostate	−66.7	−108.7
Renal, metastatic	78.3	84.0	prostate	−100.0	−22.0
Prostate	75.7	16.3	prostate	−116.7	−88.2
Prostate	65.3	74.9	breast	−140.0	−42.9
Renal	65.2	90.0	prostate	−150.0	−100.0
Breast	16.7	−46.7	lung	−166.7	−6.1
Breast	14.7	33.3	prostate	−173.2	−203.8

Data excluded several cases of aggressive tumors when the changes in CRP and tumor markers were higher than 300%.

## Discussion

Intravenous vitamin C, as administered by slow infusion, allows for plasma ascorbate concentrations an order of magnitude beyond those attainable with oral administration. Rationales for IVC therapy include preferential toxicity of ascorbate toward cancer cells [22,45], potential benefits of ascorbate for immune cells, and ascorbate inhibitory effect on angiogenesis [32,33]. In experiments with a guinea pig tumor model, tumor growth was significantly reduced in cases where intra-tumor ascorbate concentrations reached the millimolar level [46].

Laboratory studies suggest that, at high concentrations, ascorbate does not interfere with chemotherapy or irradiation and may enhance efficacy in some situations [47-53].

Vitamin C was first suggested as a tool for cancer treatment in the 1950’s: its role in collagen production and protection led scientists to hypothesize that ascorbate replenishment would protect normal tissue from tumor invasiveness and metastasis [10,11]. Cameron and Pauling observed fourfold survival times in terminal cancer patients treated with intravenous ascorbate infusions followed by oral supplementation [54]. However, two randomized clinical trials with oral ascorbate alone conducted by the Mayo clinic showed no benefit [55,56]. Most research from that point on focused on intravenous ascorbate.

Meta-analyses of clinical studies involving cancer and vitamins also conclude that antioxidant supplementation does not interfere with the efficacy of chemotherapeutic regiments [57-59].

The present manuscript provides information on how inflammation and tumor burden can affect the peak plasma ascorbate concentration achieved via IVC therapy. Both inflammation and tumor burden are representative of the total oxidative stress load of advancing cancer that IVC can help to safely reduce.

Data presented above indicate that large doses given intravenously may result in maximum plasma concentrations of roughly 30 mM, a level that has been shown to be sufficient for preferential cytotoxicity against cancer cells [22]. This is in contrast to the effects of oral ascorbate supplementation, as data according to study [60] showed that once oral intake of vitamin C exceeded 200 mg administered once daily, it was difficult to increase plasma and tissue concentrations above roughly 200 μM.

Patients with advanced or metastatic cancers demonstrate higher levels of oxidative stress and inflammation as seen in the subjects we reported in this paper. The inflammatory microenvironment of cancer cells leads to increasing oxidative stress, which apparently depletes vitamin C, resulting in lower plasma ascorbate concentrations in blood samples post IVC infusion. Another explanation for this finding may be that cancers are themselves more metabolically active in their uptake of vitamin C, causing subjects to absorb more of the vitamin, and as a results show lower plasma ascorbate concentrations in blood post IVC infusion.

The presence of metastases demonstrates an even higher demand for vitamin C due to one or the other, or perhaps both of these two explanations for low vitamin C in advancing cancer.

Many tumors in vivo appear to be under persistent oxidative stress. [61,62]. Tumor cells may overproduce ROS because the NADPH-oxidase is regulated by the GTPase Rac1, which is itself downstream of the proto-oncogene Ras [63]. Sub lethal oxidative stress promotes cell proliferation in vitro, with both superoxide and hydrogen peroxide stimulating growth [64]. Proliferation in response to hydrogen peroxide may be due to the activation of mitogen-activated protein kinases (MAPKs). Oxygen radicals augment tumor cell migration, increasing the risk of invasion and metastasis, as the p38 MAPK is activated by oxidative stress [65], and the phosphorylation of heat shock protein-27 by p38 MAPK has been shown to induce changes in actin dynamics [66]. These oxygen radicals may deplete vitamin C in cancer patients, and may contribute to the lower plasma ascorbate levels attained by IVC in cancer patients, compared to plasma ascorbate levels attained in healthy subjects.

The finding of decreased plasma ascorbate levels in cancer patients may relate to the molecular structure of ascorbic acid; in particular, the similarity of its oxidized form, dihydroascorbic acid, to glucose. Since tumor have increased requirement for glucose [67], transport of dehydroascorbate into the cancer cells via glucose transport molecules and ascorbate through sodium-dependent transporter may be elevated [68,69]. Increased accumulation of ascorbic acid in the tumor site was supported by measurements of the level of ascorbic acid in tumors in animal experiments [46]. Also, patients with advanced malignancies may have lower level of ascorbic acid in tissue, creating a higher demand for the vitamin C.

IVC therapy appears to reduce CRP levels in cancer patients. CRP concentrations directly correlate with disease activity in many cases and can contribute to disease progression through a range of pro-inflammatory properties. Being an exquisitely sensitive marker of systemic inflammation and tissue damage, CRP is very useful in screening for organic disease and monitoring treatment responses [70].

While a variety of factors can affect CRP levels, including sex, body mass index, and cardiovascular health, recent studies indicate its importance in malignant diseases. For example, increases in CRP concentrations have been associated with poorer prognosis of survival in cancer patients, particularly with advance disease independent of tumor stage [71]. According to Figure 4 above, patients with severely elevated CRP levels attain plasma ascorbate concentrations after IVC infusions that are only 65% of those attained for subjects with normal CRP levels. More detailed analysis of patients treated by IVC with follow-up several year showed that suppression of inflammation in cancer patients by high-dose IVC is feasible and potentially beneficial [42]. Inflammation plays a key role in tumor development, affecting tumor proliferation, angiogenesis, metastasis, and resistance to therapy [72-77]. Cancer-related inflammation accompanied by leukocyte infiltration, cytokine build-up, tissue remodeling, angiogenesis, and inflammatory microenvironment [78], is a key component in tumors of epithelial origins [79]. While immune cells may repress tumor growth in some cases [80,81], the inflammatory microenvironments within tumors can facilitate cancer development. In clinical studies, the use of anti-inflammatory agents is associated with reduced instances of certain cancers [82].

Inflammation is a marker of high cancer risk, and poor treatment outcome [83,84]. The subjects with highly elevated CRP concentrations have a three-fold elevation “all-cause” mortality risk and a twenty-eight fold increase in cancer mortality risk [85].

The properties of ascorbic acid as antioxidant and an enhancer of immune function, as well as the correlations between ascorbate depletion in cancer patients and prognosis [16], suggest that vitamin C may have a beneficial effect on inflammation in cancer patients. The data presented above support this idea.

## Conclusions

In summary, the data detailed in the present manuscript indicate that, while potentially therapeutic plasma ascorbate concentrations can be achieved with IVC, levels attained will vary based on tumor burden and degree of inflammation. Evidence suggests that IVC may be able to modulate inflammation, which in turn might improve outcomes for cancer patients. IVC may serve as a safe, adjunctive therapy in clinical cancer care.

## Consent

Written informed consent was obtained from the patient for the publication of this report and any accompanying images.

## Competing interests

The authors have no competing interests. The authors have no direct financial interest in the subject matter discussed in the submitted manuscript. None of the authors are employees or consultants to the organizations providing support. Additionally, the authors as the team of Riordan Clinic research group do not have any other nonfinancial conflict of interest.

## Authors’ contributions

MN, CJ analyzed data and interpreted results of analysis. MN, CJ, NR and RH conceptualized the manuscript. All authors read and approved the final manuscript.
